# Extraction of Biomolecules from Coffee and Cocoa Agroindustry Byproducts Using Alternative Solvents

**DOI:** 10.3390/foods14030342

**Published:** 2025-01-21

**Authors:** José Pedro Zanetti Prado, Rodrigo Corrêa Basso, Christianne Elisabete da Costa Rodrigues

**Affiliations:** 1 Laboratório de Engenharia de Separações (LES), Departamento de Engenharia de Alimentos (ZEA), Faculdade de Zootecnia e Engenharia de Alimentos (FZEA), Universidade de São Paulo (USP), Pirassununga 13635-900, São Paulo, Brazil; jprado1302@usp.br; 2 Instituto de Ciência e Tecnologia, Universidade Federal de Alfenas (UNIFAL), Poços de Caldas 37715-400, Minas Gerais, Brazil; rodrigo.basso@unifal-mg.edu.br

**Keywords:** coffee cherry husk, parchment skin, silver skin, cocoa bean shell, biobased solvents, supramolecular solvents, NADES, alternative extraction technologies

## Abstract

Coffee and cocoa agribusinesses generate large volumes of byproducts, including coffee husk, coffee pulp, parchment skin, silver skin, and cocoa bean shell. Despite the rich composition of these materials, studies on biomolecule extraction with green solvents are still scarce, and further research is needed. Extraction methods using alternative solvents to obtain biomolecules must be developed to enhance the byproducts’ value and align with biorefinery concepts. This article reviews the compositions of coffee and cocoa byproducts, their potential applications, and biomolecule extraction methods, focusing on alternative solvents. The extraction methods currently studied include microwave-assisted, ultrasound-assisted, pulsed electric field-assisted, supercritical fluid, and pressurized liquid extraction. At the same time, the alternative solvents encompass the biobased ones, supercritical fluids, supramolecular, ionic liquids, and eutectic solvents. Considering the biomolecule caffeine, using alternative solvents such as pressurized ethanol, supercritical carbon dioxide, ionic liquids, and supramolecular solvents resulted in extraction yields of 2.5 to 3.3, 4.7, 5.1, and 1.1 times higher than conventional solvents. Similarly, natural deep eutectic solvents led to a chlorogenic acid extraction yield 84 times higher than water. The results of this research provide a basis for the development of environmentally friendly and efficient biomolecule extraction methods, improving the utilization of agricultural waste.

## 1. Introduction

The global production of coffee beans and cocoa beans in the 2023–2024 crop season reached values of approximately 10.15 million and 4.3 million tons, respectively [1,2]. These agricultural commodities, which are widely produced and traded, generate large volumes of waste annually [3], and these byproducts are often discarded incorrectly. Such practices have aroused interest in studies aiming to use these materials for the extraction of bioactive minority compounds (BCs) [4,5], lipids [6], and macronutrients such as proteins [4,7].

Dry and wet processing are the two primary methods to obtain green coffee, the product before roasting. In dry processing, coffee fruits (cherries) are dried to a moisture content of approximately 10%. Subsequently, the dried fruits undergo mechanical dehulling to separate the coffee beans. Coffee husk is generated as a byproduct comprising the outer skin (cherry husk), pulp, parchment, and a portion of the silver skin. This husk fraction, consisting of skin, pulp, mucilage, and parchment, constitutes 40–50% of the dried fruit. In wet processing, coffee fruits are separated by immersion in water. Subsequently, the pulp and outer skin (cherry husk) are mechanically separated. After this step, the coffee seed is covered by the parchment layer, which is, in turn, coated with mucilage, a thin layer rich in pectin. In the subsequent step, the coffee beans undergo fermentation, during which the mucilage is broken down. The coffee beans are washed following fermentation to remove residual pulp material and mucilage. The final two steps before roasting are drying and dehulling, producing green coffee beans [8]. Coffee residues are mostly generated in the bean drying, dehulling, and roasting stages and are currently used for fertilizing coffee plantations and animal feed [9]. The coproducts generated represent approximately 50% of the coffee fruit, equivalent to approximately 5 million tons of waste generated annually [10]. Cherry husk (CH), coffee pulp (CP), parchment skin (PS), and silver skin (SS) are the main processing residues generated up to the bean roasting stage [11]. Research has demonstrated the feasibility of using these residues to produce biobased fuels [8] and extract BCs [12,13].

The main ingredient used to manufacture chocolate and its derivatives is cocoa nibs, obtained by processing cocoa beans. This process can be separated into two stages: preprocessing, carried out by the cocoa producer, and industrial processing. Preprocessing comprises harvesting, pod opening (where the seeds containing the cocoa nibs are separated), fermentation, drying, and storage. In industrial processing, after the cleaning step, the seeds can go directly to roasting or undergo thermal treatment, depending on the industry. In the next step, the seeds are broken, shelled, and winnowed, resulting in the extraction of cocoa nibs [14]. The cocoa bean shell (CBS) is a residue generated in the production of cocoa nibs and is obtained during the peeling of dry or roasted beans. This residue is used as fuel in boilers, as animal feed, and as fertilizer [14,15]. However, this coproduct, which represents 10–17% of the bean weight, is rich in proteins, lipids, and BCs and can be put to better use, contributing positively to the cocoa value chain [14,16].

The use of agroindustry residues as an inexpensive and sustainable source of biocompounds can reduce the negative impacts of the production and processing of coffee and cocoa. Ruesgas-Ramon et al. [17] reported that waste can damage the environment when poorly managed and discarded, causing deterioration of the landscape, the generation of bad odors, and the appearance of fungi that harm crops and human health. Additionally, the reuse of these residues has become attractive because they are rich in several BCs, such as phenolic compounds, tannins, carotenoids, lipids, and proteins, which are important inputs for the production of cosmetics, foods, and drugs [7,14]. The extraction of these compounds can be considered the most critical step for their use, requiring the use of solvents that allow good extraction yield [17].

Efthymiopoulos et al. [18] compared 12 solvents (alkanes and alcohols, along with dichloromethane and toluene) for lipid extraction from coffee grounds. The authors reported that the use of nonpolar solvents such as hexane resulted in a higher extraction yield than the use of alcohols such as ethanol. Hexane is the most widely used solvent for this process because of its high extraction capacity, low cost, high stability, and ease of recovery. However, many studies have focused on the total or partial replacement of hexane because of its high flammability and toxicity. Studies indicate the potential of hexane to cause neoplasms [19,20] and cause losses in some motor functions due to exposure for long periods [21]. These observations led to the introduction of stricter regulations by the European Union, which classified hexane as a neurotoxic substance with suspected reproductive toxicity [22].

Extraction of phenolic BCs is considered challenging because they can be unstable, and their biological activity can be easily affected by process conditions (extraction time and temperature) and the presence of light and oxygenation [23]. The most commonly used method for proteins is extraction with water or saline solution under alkaline conditions, under which most proteins have high solubility. This method has high extraction yields and is inexpensive and easy to perform, but high concentrations of alkaline solutions can cause the Maillard reaction, affecting the main characteristics of the proteins [24]. Therefore, the choice of a suitable solvent for the extraction of BCs and macromolecules is extremely important, i.e., solvents that can be used in reduced proportions relative to the solid matrix, for a short time, and at moderate temperatures without degrading the molecules of interest.

Some classes of alternative solvents, such as supercritical fluids, supramolecular solvents, and natural eutectic solvents, have demonstrated a high capacity for the extraction of BCs, lipids, and proteins in reduced amounts of time, using low amounts of solvents relative to the solid matrix, and at moderate temperatures [25].

Green solvents are intended to replace or eliminate solvents harmful to health and the environment. In addition, they can be obtained from natural and renewable sources, are nontoxic and biodegradable, are easily recoverable, and have high efficiency [26]. The use of green solvents is in line with the Sustainable Development Goals (SDGs) proposed by the United Nations. Concerning SDG 3 (Well-being and Health), the use of green solvents would reduce exposure to toxic chemicals in industrial processes; in the case of SDG 9 (Industry, Innovation, and Infrastructure), the adoption of green solvents would promote the development of innovative and more efficient extraction processes. With regard to SDG 13 (Action Against Global Climate Change), green solvents would replace solvents derived from fossil raw materials [27]. In this context, the specific characteristics of the solvents are linked to the extraction of commercially and industrially valuable biomolecules from agroindustrial residues in an economically and environmentally friendly manner without posing health risks. The solvents to be studied, as substitutes for those conventionally used in extraction processes, must be easily recoverable, selective for the target compound to be extracted, non-fossil-based, nontoxic, and capable of being used in smaller quantities in extraction processes with higher yields and greater efficiency than those using conventional solvents.

In published studies, different technologies have been used for extracting components from coffee and cocoa residues. Ruesgas-Ramon et al. [17] analyzed the extraction of biomolecules, such as phenolic compounds, fibers, and alkaloids, from these residues using natural eutectic solvents based on choline chloride. The authors concluded that the use of this class of solvents allowed high extraction yields with short extraction times and with a low volume of solvent relative to the solid matrix. Soares, Okiyama, and Rodrigues [28] studied the extraction of BCs and lipids from CBS using ethanol and isopropanol as solvents; they reported that it is possible to obtain high yields (30% for ethanol and 70% for isopropanol) from the simultaneous extraction of lipids and BCs. Silva et al. [29] used different extraction methods to obtain BCs from CH using ethanol, water, and an ethanol–water solution (1:1 *v*/*v*) as the solvent. The authors reported that the use of hydroalcoholic solution resulted in a high extraction yield of BCs, expressed as the chlorogenic acid equivalent (CGA) (97.89 mg CGA/g of extract).

Based on the above, there is a need to evaluate the use of solvents that have been studied to extract biomolecules from byproducts of coffee and cocoa processing with adequate yields and to respect the principles of green chemistry. The processes of extracting the components of coproducts of coffee and cocoa processing that lead to decreased environmental impact should also be analyzed. Therefore, the present study investigates the application of alternative solvents and technologies for extracting biomolecules from coffee and cocoa residues.

## 2. Coffee and Cocoa Agroindustry Residues

The generation of waste in agribusiness has increased in recent years due to the high demand for food, driven by population growth, making the search for reuse, recovery, and repurposing of these wastes essential [30]. Therefore, the valorization of agroindustrial byproducts has gained increasing interest because of the high abundance of components such as lipids, proteins, fibers, and BCs [3].

When poorly managed, agroindustry byproducts can generate biotic components that contaminate plantations or degrade the environment, impacting human health and the economy [17,31]. The main cause of pollution from agricultural residues is waste, underutilization, a lack of awareness, and a lack of treatment during disposal. Recently, the conversion of agricultural residues into value-added byproducts has attracted interest because of the growing market demand for products with natural additives and stricter environmental regulations [30].

The circular economy is considered an alternative to the linear production model, aiming to transform disposable goods, such as agroindustry waste, into reusable products, especially as matrices in applications of technological interest and/or high added value [32], resulting in the reduction in discarded waste and the maximization of the resources obtained.

The concept of biorefinery, with the aim of putting agroindustrial waste to better use, is in line with the principles of the circular economy. Biorefinery is based on the principle of “zero waste”, which aims to maximize the use of biological raw materials. Initially, the biorefinery concept was used only for the production of energy (ethanol, hydrogen, and butanol). Over the years, studies have shown that this concept can be extended considerably further, including the production of fertilizers, animal feed, enzymes, and proteins and the extraction of BCs [31].

Beltrán-Ramírez et al. [33] analyzed the commercial value of products derived from agribusiness residues. The authors reported a growing interest by the food and pharmaceutical industries in the extraction of carotenoids, flavonoids, and anthocyanins and in compounds that improve the nutritional and/or technological value of the formulated products, such as adding flavor or attributing properties of interest. The authors also emphasized that the use of agribusiness coproducts could reach USD 46.7 billion in consumer goods, in addition to USD 1.12 billion in functional food products by 2025. These compounds have attracted the interest of researchers and industry, demonstrating that when analyzed within the biorefinery context, agroindustrial plant wastes are a profitable and ecological option for a growing commercial market, with high potential for application in new sectors.

### 2.1. Coffee

The coffee agroindustry has high global economic importance, and coffee beans are widely traded on stock exchanges as commodities and sold worldwide. Brazil is the world’s largest exporter of coffee, producing approximately 4.2 million tons of beans in 2023–2024 crop years, followed by Vietnam and Colombia. Together, these three countries account for approximately 63.43% of the world’s production [2]. This agroindustry accounted for approximately 309 billion dollars in 2023, and 39.34% of the beans were exported to the European Union, mainly to countries such as Germany, Italy, and France; 20.40% were exported to the United States of America, and 5.60% were exported to Japan [2,9].

Owing to high global coffee consumption, the impacts of the coffee agroindustry have been evidenced in several studies that have focused on the recovery of waste generated by this sector. Bondam et al. [11] reported that approximately 23 million tons of residue are generated annually by the coffee agroindustry in Brazil (the largest producer) through the processes of peeling and mucilage removal to obtain the product of greatest interest, the bean. After harvest, coffee is classified according to weight, size, and shape and can be processed in two ways, dry and wet, with dry coffee being the most commonly used method because it is simpler and better for the environment, generating less solid and liquid waste [9]. Coffee cherries are most often dried in the sun [11] and then cleaned and peeled, which leads to the formation of CH, CP, and PS residues, the latter being the film that covers the coffee bean. SS is obtained from the roasting stage, in which the green bean is subjected to a temperature close to 200 °C to yield the roasted coffee bean [9].

The chemical composition of the residues from the coffee processing stages is variable, depending on the method by which they were obtained (wet or dry), the climate, the soil composition of each location, and the nutrients provided to the plant [34]. Several studies have investigated the composition of residues, and the results are shown in Table 1. Most residues are excellent sources of various biomolecules, such as phenolic compounds, caffeine, proteins, and fibers [8]. Bessada et al. [35] highlighted the differences in the composition of coffee residues and analyzed the countries from which they were obtained, including Brazil, India, Indonesia, Cameroon, and Vietnam. The compositions of the residues, such as the amount of caffeine, differed greatly. For example, samples from India contained 676 mg caffeine/100 g SS, whereas samples from Brazil contained 1210 mg caffeine/100 g SS.

Clark and Macrae [36] highlighted some factors, such as cultivation conditions, like climate and soil, the originating species of the residues (Arabica or Robusta), the degree of coffee roasting, and the methods used for compound extraction and analysis, which play a significant role in shaping the chemical composition of the residues. For instance, the roasting process partially degrades proteins in the dry matter, significantly influencing the final composition.

CH represents 12 to 18% of the weight of coffee cherries on a dry basis [36] and consists of a large number of fibers, such as cellulose and lignin, in addition to proteins. Owing to the high percentage of fibers, this residue can be used in the ceramics industry as a source of replacement for feldspars [34]. Owing to the high number of proteins, CH can act as plasticizers for polymer matrices and facilitate the processing of composites [34]. CH is rich in BCs and can be used in food and pharmaceutical products because of its high composition of tannins and caffeine. Soares et al. [37] studied the solid-state fermentation of CH, adding different concentrations of glucose that resulted in the production of banana and pineapple flavors, as an effort to add a fruity flavor to foods. Furthermore, the authors reported that the CH provided several components necessary for fermentation and biofuel production, such as proteins, carbohydrates, BCs, and minerals. Due to its considerable protein content and nitrogen, CH can be used as a fertilizer and for silage quality improvement [34]. Other applications for CH are presented in Table 2.

**Table 1:** Characterization of agroindustrial coffee and cocoa byproducts.

Byproduct	Composition								References
	Protein (g/100 g) ^a^	Lipids (g/100 g) ^a^	Dietary Fiber (g/100 g) ^a^	Cellulose (g/100 g) ^a^	Lignin (g/100 g) ^a^	Total Phenolic Compounds (mg GAE/g Byproduct) ^b^	Caffeine (mg Caffeine/g Byproduct)	Moisture (g/100 g)	
CH	6.6–11	0.5–3	26–32	16–43	6–24.5	4.55–10	1.3–9.8	5.7–11.98	[8,11,34]
CP	4–16.2	1–2.9	12–24	10–63	14.3–31.5	0.56–4.53	18.6–31	5–11.6	[10,11,38,39,40]
PS	0.4–3.1	0.3–0.9	89–92	40–60	25–35	2.28–2.84	1.45–58	7.6–11	[10,11,34,41,42]
SS	12–20	2.1–5.8	54–74	10–23.8	17.8–30.2	3–17.3	10–36	4–7	[10,11,34,35,43,44]
CBS	9–18.1	2–21	61–65.58	14–35	32.41–40.2	4.6–6.9	1.2–1.44	4.7–10.1	[3,4,14,28,32]

^a^ Composition in dry basis. ^b^ GAE = gallic acid equivalent. CH = cherry husk; CP = coffee pulp; PS = parchment skin; SS = silver skin; CBS = cocoa bean shell. Note: the reader is advised to consult the individual references for further information regarding analytical methods, sample preparation, and treatments during analysis.

**Table 2:** Coffee and cocoa byproducts applications.

Byproduct	Current/Potential Uses	References
CH	Composting and vermicomposting production; Production of biofuels, ethanol, and biogas; Production of mushrooms, energy drinks, and energy bars; Application in polymers and ceramics; Food for cows, pigs, and fish; Production of silage and soil conditioners; Studies in fermentation processes as a substrate; Combustion; Extraction of bioactives: carotenoids, chlorogenic acids, and other polyphenols.	[5,29,34]
CP	Fermentation as a substrate; Fertilizer; Mushroom production; Animal feed; Biofuel production; Pellet manufacturing.	[5,11,34]
PS	Pyrolysis; Extraction of bioactive; Fermentation as a substrate and support for enzyme immobilization; Production of biofuels and biogas; Production of panels in civil construction.	[34,42]
SS	Source of fiber in functional products; Color modifier in food; Functional beverages; Fertilizers; Energy source in boilers; Obtaining ethanol and methanol.	[5,11,34]
CBS	Fertilizer; Biogas; Production of polymeric films; Production of functional beverages; Extraction of bioactive; Formulation of cakes, cookies, chocolates, and beverages; Fuel in boilers; Animal feed.	[3,14,15]

CH = cherry husk; CP = coffee pulp; PS = parchment skin; SS = silver skin; CBS = cocoa bean shell.

CP is the residue generated in the largest amount, representing approximately 40% of the weight of the cherry [34]. It is rich in proteins and BCs, such as caffeine, phenolic compounds, chlorogenic acids, and tannins. CP can be used in several industries, such as food, pharmaceutical, and cosmetics [45] (Table 1 and Table 2). Due to its high organic matter content, it can be considered a promising raw material for various microbial processes or as a fertilizer [34]. In addition to the factors mentioned above, such as origin, climate, and soil type in cultivation, the pulping method strongly influences the composition of CP [36]. However, its composition is similar to CH, as both originate from the same part of the coffee cherry [34]. According to Klingel et al. [9], CP can be used to manufacture juices and jellies because of its sweet taste and BC content. In addition, CP can be used in functional antioxidant drinks containing caffeine and polyphenols. Because of the high concentrations of BC and fibers, CP flour can be incorporated into various bakery products, such as breads, cookies, muffins, brownies, spreads, and sauces (Table 2).

PS is a product generated in the coffee cherry peeling stage, representing approximately 6% of the cherry [34]. This residue is rich in fiber (approximately 90% of the composition) and is used in the formulations of new functional foods [8]. PS flour was tested in cookie formulations owing to its high insoluble dietary fiber content, which led to a significant increase in the product’s fiber content [41]. Owing to its reduced composition of proteins and lipids, this residue is used in fermentation processes, in the generation of biofuels, and in pyrolysis processes (Table 2) [44]. Thus, analyzing its chemical composition, PS is a promising source of lignocellulosic materials. Although studies on PS are relatively recent in exploring its use as a food ingredient, coffee parchment’s technological functionalities (water and oil retention capacities, water absorption, swelling, and gelation capabilities), as well as high levels of BC and fiber, open up possibilities as a physiological and technological functional ingredient in food applications and as an active component in food packaging [11,42].

SS is extracted from green coffee beans during the roasting stage, constituting 4.2% of the beans [43]. It is a product that has attracted great industrial interest because of its low moisture content and great potential for BC extraction. SS is rich in proteins, phenolic compounds, and caffeine and is an attractive product for producing functional foods, beverages, and drugs [7] (Table 1). This material has been applied in the formulation of functional yogurts due to their BC levels, with improvements in the bioaccessibility of caffeine and 5-caffeoylquinic acid (5-CQA) [46]. Blinová et al. [43] also highlighted that this coproduct could be used in fermentation as a support and source of nutrients during the production of fructofuranosidase and fructooligosaccharides by the fungus Aspergillus japonicus because of its levels of proteins and phenolic compounds. Furthermore, SS can be used in cosmetics and dermaceuticals due to its powerful antioxidant capacity. SS can be employed in producing biofuels and other industrial fields (Table 2).

### 2.2. Cocoa

The cocoa bean is the main raw material used in the production of chocolate. However, this is not its only use, as it can also be used in the production of cocoa powder and cocoa butter, with numerous applications in the food and cosmetics industries, among others. The world production of cacao fruit in the 2023–2024 harvest was approximately 4.3 million tons [1], occurring in countries with tropical climates, such as Côte d’Ivoire, Ghana, Indonesia, and Brazil [1,15].

CBS is one of the main residues generated, representing 12 to 20% of the seed [14]. CBS is obtained by dehulling dry cocoa beans; the cotyledon shells are separated to obtain the product of greatest interest, which is used in the form of nibs for the production of chocolate. The husks can also be obtained in the thermal treatment stage, in which the seeds are roasted, followed by separation of the husks and the cotyledon [14].

Approximately 700 thousand tons of CBS are estimated to be generated annually and can be used for nonfood purposes, such as in boilers for energy generation, in the production of fertilizers, and for other purposes [15] (Table 2). However, the application of these residues for purposes where their rich constitution of components of nutritional, pharmaceutical, and/or technological interest are not used can be considered an underutilization of this coproduct. CBS is rich in proteins and BCs, such as caffeine, phenolic compounds, and theobromine (Table 1). They can be used in the formulation of functional foods, such as cereal bars [47], and in bakery products because these residues, when transformed into flour, have a flavor close to the original flavor, increasing nutritional benefits [14,48]. Okiyama et al. [14] reported that CBS has a lower lipid content and a fiber content three times greater than that of nibs.

Studies have shown that the lipids obtained from CBS may be good substitutes for cocoa butter because they have similar fatty acid compositions [28]. The same authors also reported that CBS can be used in the formulation of functional foods owing to the properties of its proteins and because it is rich in various BCs, such as phenolic compounds and caffeine [28,48].

## 3. Extraction of the Components of Interest from Coffee and Cocoa Residues

To be better used and because they are rich in biocompounds, such as proteins, lipids, fibers, and phenolic compounds, the CH, CP, PS, and SS of coffee, along with CBS, are being evaluated as matrices for the extraction of these biomolecules. To present a more sustainable and profitable way to use these residues, the biorefinery concept can be used [15,17].

Solid–liquid extraction is a technique widely used for the recovery of the aforementioned compounds. It can be applied to different matrices, with a wide variety of solvents, and when combined with agitation and heating, allows acceptable extraction yields to be obtained [11]. The most common disadvantage associated with traditional solid–liquid extraction methods is the need for prolonged periods of contact between solids and solvents, together with the use of temperatures higher than room temperature. These parameters, which are often necessary for some systems, can lead to degradation of the compound to be extracted [23].

The choice of an ideal solvent or method in the biomolecule extraction stage can lead to high yield values, reducing the extraction time and allowing intact extracts to be obtained. The choice of solvent is directly associated with the characteristics of the component to be extracted so that the solvent is able to bind to the solute to be extracted, making the use of each solvent specific for each type or group of molecules [49,50].

### 3.1. Principles of Biomolecule Extraction Processes

The extraction of proteins, lipids, phenolic compounds, and carotenoids, among other compounds, is highly important for the chemical and food industries. The primary objective of this process is to obtain the compounds of interest with intact integrity and functionality from a matrix of biological origin. Conventional extraction methods, such as maceration, demand large amounts of solvent, energy, and time [51]. Among the parameters that directly influence the yield of biomolecule extraction by conventional methods are temperature, agitation, type of solvent, and pretreatment of the material to be subjected to extraction [51].

Temperature is a critical parameter for biomolecule extraction methods. High temperatures tend to reduce the viscosity of the solvent and extract, increasing the diffusivity of the target component and thus facilitating mass transfer. An increase in temperature also favors the solubility of the solute in the solvent. However, the use of high temperatures can lead to the degradation of BCs and proteins, altering their molecular structure and consequently causing losses in their bioactive properties [23].

Pretreatment of the solid matrix is necessary to improve the extraction yield of a target compound; such pretreatment methods include increasing the surface area to be exposed to the solvent and removing water. Such procedures are performed to make the biomolecules more accessible to the solvent, thus increasing the mass transfer and consequently the extraction yield [52].

Another key factor for the effectiveness of an extraction process is the agitation of the medium, which prevents sedimentation, ensuring uniform contact between the solid matrix and the solvent. Adequate agitation also accelerates diffusivity and ensures greater homogeneity of the solution temperature [51,53].

New techniques have been developed to improve the extraction efficiency of biomolecules, preserving the integrity of BCs, using a smaller volume of preferably nontoxic solvents, and increasing energy efficiency [54]. Alternative extraction methods include microwave-assisted extraction (MAE), ultrasound-assisted extraction (UAE), pulsed electric field-assisted extraction (PEF), pressurized liquid extraction (PLE), and supercritical fluid extraction (SFE).

MAE uses electromagnetic waves, in the frequency range of 0.3–300 GHz, to rotate the dipoles of the molecules and promote ionic conduction, causing heating and disintegration of the cell wall of the solid matrix and facilitating the release of intracellular BCs [23]. Compared with conventional extraction, MAE causes a rapid increase in the local temperature, enabling shorter extraction times and reduced amounts of solvent compared to in solid extraction [55]. According to authors such as Azmir et al. [54] and Osorio-Tobón et al. [23], MAE has a more significant impact on polar BCs, such as phenolic compounds, as these strongly absorb microwave energy, facilitating ionic conduction and dipolar rotation. The electromagnetic waves are composed of two perpendicular oscillating fields, which result in a higher extraction yield following three steps: The first consists of the separation of the BCs from the active sites of the sample caused by the electromagnetic waves. In the second step, solvent diffusion occurs in the sample matrix, which has undergone cellular ruptures caused by the method. Finally, the mass transfer of the BCs to the solvent occurs [54]. The method’s main advantages are the low amount of solvent used, the reduction in thermal gradients, high extraction yields, and a selective technique for extracting organic compounds and BCs [54,55]. However, the most significant disadvantage of MAE is the limited compatibility of materials and solvents, since not all solvents or matrices are suitable for interaction with microwaves. Nonpolar solvents, for example, have a low capacity to absorb microwave energy, which can limit heating efficiency and, consequently, extraction yield [23,56].

UAE consists of the use of ultrasonic waves at frequencies of 20–100 kHz to produce cavitation [23]. This process is highly energetic and consists of a series of compression and decompression cycles in the liquid medium, generating a change in pressure and the formation of bubbles [56]. When these bubbles collapse, they produce very intense shear forces that propagate in the medium, instantly increasing the local temperature to up to 5000 °C at a pressure of 200 MPa, similar to a shock wave. This cavitation phenomenon can cause destruction of the matrix cell walls, allowing the solvent to come into contact with the intracellular components and increasing mass transfer [10,56]. Small volumes of solvent can be used in UAE, but the main disadvantage is that cavitation can cause the degradation of BCs [23]. The equipment used for UAE can vary, with the most basic and accessible being ultrasonic baths. At the same time, there are systems with probes that are more powerful and adaptable, providing a greater concentration of ultrasonic energy. The main components include a power generator, transducer, amplifier, and probe, which convert electrical energy into acoustic waves. Crucial parameters, such as ultrasound power and amplitude, directly influence the extraction yield, increasing solvent penetration and releasing more phenolic compounds [56]. This method allows high extraction and recovery yields of BCs in a shorter process time and can use smaller volumes of solvents, working efficiently with GRAS solvents. In addition, the UAE can be adjusted to meet different types of BCs, whether polar or nonpolar, depending on the extraction conditions (such as solvent, temperature, power, and ultrasound amplitude). However, it is necessary to balance power, temperature, and time to avoid degradation of the target compounds [23,54,55].

Wen et al. [13] investigated the extraction of proteins from dried flakes of coffee silver skin using the MAE and UAE techniques, comparing them with the conventional alkaline extraction technique. The authors reported yields for 10 min of extraction of 43.53% for MAE, 14.04% for UAE, and 6.23% for conventional extraction. They suggested that MAE achieved a higher extraction yield due to microwave irradiation, which causes direct heating within the residue matrix, raising the local temperature and pressure, which improves mass transfer.

Another method used is PEF, which consists of applying a high-voltage electromagnetic pulse (1–80 kV/cm) for a short period to cause damage to the cell wall of the solid matrices, making the cells permeable to the solvent, consequently increasing the extraction yield [32]. This method is especially effective for treating plant matrices with strong cell walls and is also useful for deactivating unwanted microorganisms, functioning as an alternative to pasteurization [32]. In addition, one of the advantages of this method is the possibility of being used for extraction at room temperature, making it a viable alternative for extracting thermolabile BCs [10]. Other advantages of PEF include increased extraction yields, reduced extraction time, and lower solvent consumption than conventional methods. As a disadvantage, the technique can favor the extraction of specific bioactives, such as flavonoids, but with less effect on others, such as tannins. In addition, the effectiveness can vary depending on the variety of raw materials and the industrial treatment applied, requiring specific adjustments for each sample [10,23,32].

Barbosa-Pereira et al. [57] applied PEF as a pretreatment to extract phenolic compounds from CBS and SS, compared with conventional extraction. The polyphenols extraction optimum conditions differed for CBS (11.99 μs PEF pretreatment time, 991.28 number pulses, 1.74 kV cm^−1^ PEF strength, water–ethanol 61:39 *v/v* solvent, and 118.54 min of extraction) and SS (5.45 μs PEF pretreatment time, 1000 pulses number, 1.37 kV cm^−1^ PEF strength, ethanol–water 63:37 *v*/*v*, and 75 min of extraction). The authors mentioned that using PEF pretreatment improved phenolic extraction by 75% for CBS and 83% for SS compared to conventional extraction, proving that PEF could damage the cell walls of the residues, leaving the BC accessible to the solvent.

PLE uses solvents at high temperatures (50–200 °C) and pressures (30–200 bar), accelerating extraction compared with conventional methods. High pressures allow an increase in the boiling temperature of the solvents so that they remain in the liquid state while inducing the wall of the solid matrix to break, increasing diffusion and mass transfer [51]. In addition, PLE uses less solvent and can use ecological solvents, such as subcritical water, making the process more sustainable. However, using high pressure and temperature requires expensive equipment and high energy demand, in addition to potentially resulting in lower extraction yields owing to the degradation of thermolabile biomolecules [23,32,58].

SFE uses solvents at temperatures and pressures above the critical point, where there is no longer any distinction between the gaseous and liquid phases. Supercritical fluids are characterized by having gas–liquid properties, such as gas-like diffusivity and viscosity and liquid-like solvation and density [59]. Their properties can be modulated according to the objective of the process in which they are applied, by changing the temperature or pressure, adding cosolvents, and even changing the solvent to be used, the most common being carbon dioxide, chlorodifluoromethane, and nitrous oxide [56,59]. The reduced surface tension and viscosity of the solvent used and a diffusion coefficient close to that of gases increase the penetration of the solvent into solid matrices and facilitate the extraction of the target compounds [56]. SFE, especially using carbon dioxide, does not involve high temperatures, which helps to avoid the thermal degradation of BCs. In addition, extraction efficiency depends on the precise management of temperature and pressure, which may require sophisticated equipment and increase operational costs [10,56,59].

Although the extraction method is linked to the yield and quality of the compounds obtained, the choice of solvent is also a determinant in this process [11]. The use of an appropriate solvent is based on several factors, such as its physical properties, cost, and toxicity. The polarity, affinity for the solute, surface tension, density, and viscosity of the extractant are some of the characteristics that must be analyzed prior to its use in an extraction process [49]. Intermolecular interactions, such as hydrogen bonding and van der Waals forces, facilitate the dissolution of compounds in suitable solvents; more polar solvents are optimal for extracting polar compounds such as phenolic compounds, and nonpolar solvents are optimal for extracting nonpolar compounds such as carotenoids [50,60,61].

Traditional solvents such as methanol and hexane are effective at extracting a wide range of compounds but may present toxicity, separation difficulties, or adverse environmental impacts. An inadequate choice of solvent can limit the efficiency of the process and affect the properties of the extracted biomolecules. Given this scenario, interest in the development and use of green solvents, such as biobased solvents, supercritical fluids, natural eutectic solvents, and supramolecular solvents, is increasing. The use of these solvents can not only reduce the environmental impact but also promote greater selectivity and efficiency of the extraction process.

### 3.2. Extraction of Biomolecules Using Conventional Solvents

The results of biomolecule extraction depend especially on the type of extraction, the choice of solvent, and the origin of the material and its specific properties [8].

The extraction of phenolic compounds from CH was analyzed by Rebollo-Hernanz et al. [62] using methanol as the solvent; they obtained promising results at 100 °C after 90 min of extraction. Silva et al. [29] obtained better results at shorter times and temperatures when ethanol–water (1:1 *v*/*v*) was used as the solvent and demonstrated the greater effectiveness of ethanol than methanol in the extraction of phenolic compounds from CH.

Macías-Garbett et al. [63] used water to extract phenolic compounds from PS and CP in sequential extractions assisted by PEF pretreatment (5 min, pulse frequency of 5 Hz, and pulse voltage of 18 kV) and MAE (540 s, 25–135 °C, 145 mbar). Before extraction trials, PS and CP were sun-dried, and the moisture values were standardized to lower than 10% (weight). The results revealed good extraction yields, with values of 321.17 mg gallic acid equivalent (GAE)/100 g PS and 1443 mg GAE/100 g CP.

Soares et al. [28] evaluated the simultaneous extraction of BCs and lipids from CBS using ethanol and isopropanol as solvents. The authors reported that the use of hydrated alcohols was adequate to extract BCs, mainly alkaloids, and that isopropanol and absolute ethanol provided the best lipid extraction yields.

Botella-Martínez et al. [3] studied the conventional extraction of phenolic compounds from CBS using water. Mellinas et al. [4] used MAE with water as the solvent (5 min, 97 °C, microwave power (500 W), stirring rate (400 rpm), heating rate (20 °C/min), and solid-to-liquid ratio of 0.04 g/mL) and obtained results superior to those obtained by Botella-Martínez et al. [3], demonstrating that MAE is a better extraction method for the production of phenolic compounds.

Aguilera et al. [64] extracted phenolic compounds from PS using a solution of methanol and water (1:1 *v*/*v*) as the solvent, whereas Mirón-Mérida et al. [42] used ethanol–water (7:3 *v*/*v*). The latter authors obtained 2.14 g of total phenolic compounds (TPCs)/kg PS, values similar to those obtained by Aguilera et al. [64] (2.42 g TPC/kg PS), with a shorter extraction time and temperature, indicating that ethanol was better for the extraction of phenolic compounds from PS.

Wen et al. [65] used UAE for the recovery of phenolic compounds from SS and obtained 8.94 mg GAE/g SS in 10 min using methanol as the solvent. Prandi et al. [7] used the same solvent in a conventional extraction process for 12 h and obtained 5.36 mg GAE/g SS. These studies show the ability of ultrasound to cause greater cell disruption and microcracks, helping increase extraction efficiency in a shorter time interval.

Table 3 shows the use of different methods and solvents for the extraction of different target compounds from the CH, CP, PS, and SS residues of coffee and from CBS. This table presents the results and the process variables studied, indicating the need for the further study of alternative solvents. In these studies, the authors conducted extraction trials using different approaches, either with residues that underwent no further pretreatment or after drying at different levels. Therefore, the readers are advised to consult individual references for additional information regarding raw material preparation.

### 3.3. Extraction of Biomolecules with Alternative Solvents

The extraction of natural compounds from agroindustry residues is already well established in several production chains, such as vegetable oils, wines, and juices and in the pharmaceutical industry [97]. Traditional solvents, such as hexane and methanol, are the most commonly used solvents in extraction processes [50]. However, most of these solvents have some limitations due to the long extraction times and the need for large amounts of solvent relative to the solid. The use of these solvents can also greatly affect the thermal decomposition of the compound of interest due to the need for high temperatures, in addition to promoting the emission of pollutants into the atmosphere and the release of toxic components during the extraction processes, adding risks to the health of workers and consumers [26].

To mitigate the problems caused by the use of conventional solvents, alternative solvents have been widely studied and have shown good results for the extraction of BCs. An alternative solvent must follow the principles of green chemistry, not release toxic compounds into the environment, present low volatility, be biodegradable, and allow extraction with low energy expenditure [26,50]. Alternative solvents can be divided into four groups: biobased, supercritical, supramolecular, and neoteric.

#### 3.3.1. Biobased Solvents

Biobased solvents are obtained from renewable sources and are generally produced in biorefineries that convert biomass into fuels, energy, and chemicals; they are considered potential substitutes for solvents of fossil origin (petroleum derivatives) [98]. These solvents have low toxicity and can be used in various industrial sectors, such as in foods, pharmaceuticals, paints, cosmetics, and household care products [26,50,98]. Some biobased solvents include alcohols, esters, glycerol derivatives, terpenes, gamma-valerolactone, furan derivatives, and furfurals [50].

In general, biobased solvents have a low viscosity, which facilitates their handling and mass transfer [50]. Biobased solvents have already been used to extract BCs from coffee residues and CBS. Kulkarni et al. [77] analyzed the efficiency of three organic solvents (ethanol, acetone, and ethyl acetate) for the extraction of phenolic compounds from SS. The results showed that ethanol extraction resulted in a greater concentration of phenolic compounds (161 mg GAE/L of extract) than did acetone (22 mg GAE/L of extract) or ethyl acetate (9 mg of GAE/L of extract) extraction. The authors reported that the viscosity and polarity of the solvent directly affected the composition of the extract obtained from SS.

Concerning the suitability of different solvents and techniques for extracting specific biomolecules, the studies compiled in Table 3 indicate that the biobased solvent ethanol, alone or in a mixture with water, is the most employed solvent to extract polar BCs, such as phenolic compounds. Silva et al. [29] analyzed how the ethanol–water solvent hydration level influenced the extraction of BCs from CH. The highest extraction yield values were obtained using a 1:1 (*v*/*v*) mixture of ethanol and water for conventional (97.89 mg CGA/g CH) and UAE (90.95 mg CGA/g CH). Aiming to optimize the hydration of ethanol to extract total phenolic compounds and caffeine from PS, Mirón-Mérida et al. [42] perform the Box–Behnken experimental design. The highest extraction yield results were obtained using ethanol–water 7:3 *v/v* (2.14 g GAE/kg PS and 1.34 g caffeine/kg PS) at 75 °C. The authors highlighted the critical interaction between the temperature of extraction and ethanol hydration, particularly for caffeine extraction.

Buyong and Nillian [79] extracted phenolic compounds from SS using conventional extraction with ethanol, water, and methanol for 30 min at 60 °C. Methanol provided the highest yield of phenolic compounds, 15.24 mg GAE/g of extract, followed by ethanol (11.2 mg GAE/g of extract) and water (9.48 mg GAE/g of extract). The authors suggested the use of ethanol because, in addition to adhering to the principles of green chemistry, the extract provided good yields of the target compounds.

Jensch, Schmidt, and Strube [84] used various pressurized solvents, such as water–ethanol (8:2 to 2:8 *v*/*v*), ethanol, methanol, isopropanol, ethyl acetate, and hexane, to extract caffeine and catechin from CBS. The results revealed that water–ethanol solvents exhibited a higher solubilization capability of the BCs than the other extraction solvents evaluated.

Okiyama et al. [86] extracted flavanols and methylxanthines from CBS with pressurized ethanol (10.35 MPa) at temperatures of 60, 75, and 90 °C. The flavanol extraction yield was similar to that obtained by Soares et al. [28] with ethanol in conventional extraction under atmospheric pressure (33 to 38%). However, the use of pressurized ethanol made it possible to obtain theobromine and caffeine extraction yields that were 1.7 to 3.3 times higher than those of conventional extraction [28,86].

#### 3.3.2. Supercritical Fluids

Carbon dioxide (CO_2_) is the most widely used supercritical fluid due to its moderate critical temperature (31.3 °C) and pressure (7.38 MPa) and recognition as a safe solvent. CO_2_ is recommended for extracting nonpolar substances; adding cosolvents, such as ethanol, can increase the solubility of polar compounds such as caffeine and phenolic compounds [11,50,58].

CO_2_ was used to extract caffeine from CH with an extraction yield of 35% [66]. Andrade et al. [70] extracted phenolic compounds from the same material using supercritical CO_2_ with or without the addition of ethanol as a cosolvent; the results showed that the addition of the cosolvent at any of the concentrations studied did not affect the results, with a maximum value of 28.1 mg GAE/g of extract obtained.

Mazzutti et al. [85] extracted phenolic compounds from CBS via three different methods: SFE using CO_2_, PLE using ethanol, and Soxhlet extraction with hexane. The best results, 7.2 mg GAE/g of extract, were obtained with pressurized ethanol, followed by Soxhlet extraction (5.62 mg GAE/g of extract) and SFE (4.02 mg GAE/g of extract). Despite having the lowest extraction yield among the methods analyzed, SFE presented a reduced extraction time (2 h) at 50 °C, yielding an extract with higher antioxidant activity and higher carotenoid content than the other methods did.

Nasti et al. [75] studied the extraction of lipids and caffeine from SS by comparing supercritical CO_2_ SFE and hexane Soxhlet extraction. The authors reported that the lipid yields increased with increasing pressure at constant temperature, as the reduction in CO_2_ density allowed greater diffusion and mass transfer between the lipids and the solvent. When comparing the extraction methods in terms of yield, SFE and Soxhlet extraction were similar, and it was possible to extract all the lipids contained in the material. The extraction time with supercritical CO_2_ was considerably shorter (2 h) than the Soxhlet extraction (6 h). With respect to caffeine, SFE resulted in higher yields (0.14 g caffeine/g SS) than those obtained with hexane (0.03 g caffeine/g SS).

#### 3.3.3. Supramolecular Solvents

Supramolecular solvents (SUPRASs) are nanostructured liquids formed by a colloidal suspension of amphiphilic components through a spontaneous process of coacervation and self-assembly [12]. An external agent, such as a change in pH or temperature or the addition of salts, can accelerate the aggregation of amphiphilic compounds [99,100], with the formation of SUPRASs occurring in two steps. In the first stage, a solution of amphiphilic compounds, containing supramolecular aggregates such as micelles or reverse vesicles, is prepared above their aggregation concentration, and the formation of these aggregates depends on the available spaces and the size of the hydrophobic chain of the amphiphile, as well as the chemical groups that come into contact with water [101]. Aggregates occur due to interactions between the hydrophobic and hydrophilic parts of the molecules, leading to the formation of stable structures. The addition of a salt or a change in the pH of the system is subsequently performed to induce coacervation, increasing the size of the supramolecular aggregates by decreasing the repulsion between the components. The growth of these aggregates causes the appearance of droplets that are associated with each other, and because they are less dense than the equilibrium solution (rich in water), they separate from this phase, forming a phase rich in SUPRASs [12].

The SUPRAS phase, which is rich in colloidal aggregates, is of great interest to the academic world and has been studied in many aspects, such as in the formation of polymers [102]; in the extraction of BCs, such as phenolic compounds, proteins, and carotenoids [12,80,101,103]; in the extraction of pesticides [100]; and in water decontamination [99].

Some studies have aimed to extract caffeine from agroindustry residues using different compositions of SUPRASs as solvents. Torres-Valenzuela et al. [80] used octanoic acid and ethanol in the formation of SUPRASs and obtained 3.6 mg of caffeine/g CP at room temperature and after 15 min of extraction. In another study, Torres-Valenzuela et al. [12] compared the efficiency of different SUPRAS compositions in caffeine extraction and obtained the best result, 3.32 mg caffeine/g coffee grounds, using a SUPRAS obtained from a mixture of 24% hexanol, 30% ethanol, and 46% water (*v*/*v*). These results were compared with those of the study by Bravo et al. [104], who extracted caffeine from coffee grounds derived from different types of beverage preparations (filter, espresso, plunger, and mocha), and the residues were previously freeze-dried and defatted and subjected to extraction with water at 90 °C. Torres-Valenzuela et al. [12] reported that extraction with SUPRASs provided similar results to extraction with water, without the need for freeze-drying and lipid extraction pretreatments, in addition to being conducted at room temperature.

SUPRASs composed of hexanol, ethanol, and water were used in the extraction of caffeine and chlorogenic acids from coffee grounds, and the results were compared to those obtained via extraction with water, conventionally or in ultrasonic baths [105]. The use of SUPRASs made it possible to obtain results similar to those of extraction with water only for caffeine (0.885 to 0.972 mg caffeine/g dry coffee grounds). In the case of chlorogenic acids, extraction with water at 50 °C provided the best results (1.15 mg CGA/g dry coffee grounds). The authors mentioned that the different behaviors of BCs may be associated with their distribution between the SUPRAS phase (which is rich in amphiphiles) and the equilibrium phase (which is rich in water). These results indicate the need for further study of the thermodynamics related to systems with supramolecular solvents.

In general, studies in the literature indicate that the functional groups of amphiphilic molecules (alcohols or carboxylic acids, for example) influence the ability to establish polar and hydrogen bond interactions with BCs, whereas the size of the carbon chain influences dispersion interactions [12]. On the other hand, amphiphiles with the same chemical function and different carbon chain sizes (octanoic and decanoic acids, for example) have similar BC extraction capacities with different compositions of organic solvents and water [12]. In fact, the shorter the carbon chain of the amphiphile is, the greater its ability to interact via hydrogen bonding, which allows for the use of a smaller amount of organic solvent in the production of SUPRAS. The types of organic solvents (ethanol and tetrahydrofuran, for example) influence the extraction of BC due to their polarities, and the authors recommend the use of ethanol because it is a biobased solvent with low toxicity [12,80].

Research by Torres-Valenzuela et al. [12,80] indicated that the BC extraction yield is a function of the increase in the concentration of amphiphilic compounds in the system, and this behavior results from the increased availability of interactions/bonds with the BCs. With respect to the amount of water in the system, Ballesteros-Gómez and Rubio [106] reported that the size of the cavities formed by hexagonal aggregates increases with the amount of water in the system, which leads to the possibility of using these solvents as liquids with restricted access, i.e., liquids that exclude or minimize the capture of macromolecules (such as proteins) and maximize the capture of micromolecules, such as BCs. It has also been reported that SUPRAS are considered solvents with high efficiency and low toxicity, and the choice of component to be used in SUPRAS synthesis directly influences the toxicity, flammability, and biocompatibility of the solvent [50,106].

#### 3.3.4. Ionic Liquids and Eutectic Solvents

The term neoteric is attributed to unconventional solvents, characterized by the possibility of adjusting their physical and chemical properties according to their composition, which allows them to be used in various applications [50]. The best-known and most-studied solvents in this class are ionic liquids (ILs) and deep eutectic solvents (DESs).

ILs are composed of aqueous solutions of salts composed of cations and anions with a melting point below 100 °C and unique physicochemical properties, such as low vapor pressure, excellent thermal, chemical, and physical stability, and excellent solubility of organic, inorganic, and organometallic compounds [50,69]. However, ILs have low degradability and low biocompatibility with macro- and micromolecules and are, for the most part, very expensive. A remarkable characteristic of ILs in the extraction of BCs is their ability to penetrate and alter the biomass cell walls, facilitating the release of compounds [50,68,69].

Román-Montalvo et al. [68] evaluated the performance of ILs composed of choline and different counterions for the extraction of caffeine from CH. Choline hexanoate presented the best results, with 4221.1 mg caffeine/kg CH, which was higher than that obtained from Soxhlet extraction with ethanol (823.9 mg caffeine/kg CH). The authors attributed the higher extraction yield to the ability of ionic liquids to interact with the alkaloid, ensuring more efficient mass transfer [68].

In 2003, DESs were presented as a new type of neoteric solvent by Abbott et al. [107], who used a mixture of urea and choline chloride, which have high melting points when pure but a much lower melting point than that of the precursors when mixed. Eutectic mixtures have important properties for extraction processes (low volatility, thermal stability, low toxicity, low flammability, and biodegradability) and are widely used owing to the liquefaction and greater solubility provided by the decreased melting point of the mixture. This is due to the formation of hydrogen bonds between the components, which always contain a hydrogen bond donor (HBD) and a hydrogen bond acceptor (HBA) [108]. When these components are derived from two or more natural products, they are called natural deep eutectic solvents (NADESs).

NADESs are considered a subclass of DESs formed, for example, by organic compounds such as amino acids, sugars, and alcohols [109,110]. The most widely used hydrogen acceptor for the formation of NADESs is choline chloride because of its low cost, nontoxicity, biodegradability, and easy synthesis and because it is considered a provitamin in Europe [108]. In addition, Yang [111] reported that the choline cation can interact with cell walls through electrostatic interactions or hydrogen bonds, leading to cell disintegration, which increases BC extraction. However, several other natural compounds can be used. Table 4 presents the main extractants and for what purpose they are considered the best choices in the context of extracting BCs from coffee and cocoa byproducts.

Frade et al. [112] and Hayyan et al. [113] suggested that the toxicity of NADESs is dependent on the composition and concentration of the solution. Compared with aqueous solutions of their pure precursors, NADESs exhibited greater toxicity, which may be related to the structures formed by the eutectic mixtures and the synergistic effects between the components. The most-accepted explanation is that the change in charge that occurs with the hydrogen interactions caused by the components of the NADES increases the toxicity of the mixture [108].

Dai et al. [97] studied several other components for the formulation of NADES, such as lactic acid, malic acid, sucrose, glucose, propane diol, and sorbitol. This study analyzed the effect of adding water in the synthesis of NADES to reduce the viscosity of the medium, since high viscosities reduce mass transfer during extraction. Another variable that was able to reduce the viscosity of the solution was an increase in temperature from 25 to 40 °C. As a result, the authors concluded that the addition of water to a NADES improved the extraction of polar compounds, whereas low amounts of water make NADESs excellent extractants of nonpolar substances.

Owing to their properties, several authors have used NADESs for the extraction of biomolecules from agroindustry residues. Ruesgas-Ramon et al. [17] aimed to extract caffeine and phenolic compounds from CBS with NADESs and analyzed six types of HBDs and HBAs. This study also compared extraction using a NADES with that using a hydroalcoholic solution (ethanol–water 7:3 *v*/*v*) and obtained higher extraction yields of phenolic compounds when ethanol was used, followed by a NADES composed of choline chloride and lactic acid. Compared with the results of the study by Jokic et al. [83], who evaluated the extraction of phenolic compounds using subcritical water, the extraction results using NADESs were superior.

da Silva et al. [73] analyzed the extraction of caffeine and chlorogenic acids from different residues of the coffee value chain (mixtures of CH and PS, in addition to CP and SS) under agitation at 60 °C for 30 min. Different compositions of hydrophilic NADES (with the addition of 10 to 20% water) and hydrophobic NADES, in addition to water, were used for comparison purposes. In the case of CP, the NADES composed of guanidinium chloride, lactic acid, and water (GuCl:LA:W, molar ratio 10:17:15) allowed the extraction of 8.88 ± 0.55 mg of caffeine/g CP, a value similar to that obtained by extraction with water (8.47 ± 0.32 mg caffeine/g CP). For SS, the results when choline chloride, lactic acid, and water (CC:LA:W, molar ratio of 1:2:2) and water alone were used were also similar (8.19 ± 0.22 and 8.04 ± 0.13 mg caffeine/g SS, respectively). However, for the extraction of chlorogenic acids, the NADES presented higher values (1.68 mg CGA/g CP) than those obtained with water (0.02 mg CGA/g CP).

A study by Vieira et al. [114] evaluated the effect of the chemical structure of HBDs on the extraction efficiency of phenolic compounds, using choline chloride as an HBA. For the analysis, the following fatty acids were used: linear monocarboxylic acids (acetic acid, propionic acid, butyric acid, and valeric acid), aromatic acids (phenylacetic acid, 3-phenylpropionic acid, 4-phenylbutyric acid, and 5-phenylvaleric acid), dicarboxylic acids (malonic acid and glutaric acid), hydroxy monocarboxylic acids (lactic and glycolic acids), 1-hydroxy dicarboxylic acid (malic acid), and 1-hydroxy tricarboxylic acid (citric acid). Monocarboxylic acids resulted in extraction yields of 23 to 34 mg TPC/g of extract, which are higher than those of the other compounds evaluated, such as citric acid (15 mg TPC/g of extract) and phenylpropanoic acid (14 mg TPC/g of extract). The authors reported that the addition of hydroxyl groups to the HBD structure led to lower extraction yields. An increase in the size of the carbon chain and the presence of a phenyl group were favorable for extraction [114]. In general, the greater the presence of -OH and -COOH groups in the solvent is, the lower the extraction yields of phenolic compounds. Moreover, the presence of water in the solution increased the mass transfer from the matrix to the solvent due to the reduced viscosity, which facilitated the extraction of some BCs, such as caffeine. However, high water addition may cause extraction to not occur properly. The optimal water addition point in the extraction was 50% for the two best DES conditions obtained (choline chloride with butyric acid and choline chloride with phenylpropionic acid). The researchers obtained valuable information that an increase in the solid/liquid ratio (5–120 g/L range) has a slight effect on phenolic compound extraction [114].

Various recovery approaches have been investigated to enhance the sustainability of DES utilization. Techniques like solvent back-extraction and anti-solvent precipitation facilitate the recovery of both DESs and BCs. Nonetheless, research on the reusability of DESs remains relatively scarce, highlighting the necessity for further exploration in this area [50].

## 4. Conclusions

Coffee and cocoa agroindustry residues have the potential for extracting different components. Coffee pulp and cocoa bean shell presented a relatively high lipid composition, whereas silver skin presented a relatively high protein content. One of the best applications of coffee husk is the extraction of caffeine, while parchment skin best allows for the production of cellulose and lignin.

The evaluation of studies focusing on the extraction of macro- and microcomponents of these residues indicates that the choice of a solvent, or a mixture of solvents at different concentrations, determines the yield of the process and must be made on the basis of the properties of the solute of interest. The extraction of phenolic compounds from these byproducts has mostly been investigated in comparison with the extraction of proteins, lipids, and fibers, indicating the need for intensified research efforts related to the acquisition of these macrocomponents.

The present review contributes to guiding the choice of solvents and the best methods for the extraction of a particular biomolecule from the residues considered. Most extraction processes focus on biobased solvents, especially ethanol, showing that research on the application of SUPRAS and NADES of different compositions is still necessary for the extraction of biomolecules from coffee and cocoa residues.

## Figures and Tables

**Table 3:** Methods for the extraction of biomolecules from coffee and cocoa agroindustry byproducts.

Byproduct	Extraction Target	Solvent	Extraction Conditions	Extraction Method	Results	References
CH	Total phenolic compounds	Methanol	90 min 100 °C	Conventional	15.99 mg GAE ^a^/g extract	[62]
	Total phenolic compounds Flavonoids	Water–ethanol (1:1 *v*/*v*)	60 min 60 °C 60 min 40 kHz 35 °C	Conventional Ultrasonic bath-assisted extraction	97.89 mg CGA ^b^/g CH 9.93 mg CE ^c^/g CH 90.95 mg CGA ^b^/g CH 15.69 mg CE ^c^/gCH	[29]
	Caffeine	Supercritical carbon dioxide (CO_2_)	300 Bar 373 K	SFE	84% caffeine extraction yield	[66]
	Carotenoids	Acetone–methanol (7:3 *v*/*v*)	30 min 55 °C	Conventional	221.35 mg β-carotene/L extract	[67]
	Caffeine	Ethanol ILs (choline hexanoate)	7 h 78 °C 37 min 70 °C	Soxhlet Conventional	823 mg caffeine/kg CH 4221.1 mg caffeine/kg CH	[68]
	Lignin	ILs (diisopropylethylammonium acetate)	4 h 120 °C	Conventional	71.2% lignin extraction yield	[69]
CH	Lipids Total phenolic compounds	Supercritical carbon dioxide (CO_2_) Ethanol Ethanol	300 Bar 323.15 K 6 h 78 °C 2 h 55 kHz 25 °C	SFE Soxhlet Ultrasonic bath-assisted extraction	1.97 g extract/g CH 28.1 mg CGA/g extract 4.8 g extract/g CH 151 mg CGA/g extract 3.1 g extract/g CH 133.4 mg CGA/g extract	[70]
	Caffeine Total phenolic compounds	Water	100 °C 5 min 900 W 5 min	Conventional MAE	1.64 µg caffeine/mL extract 130.39 µg GAE/mL extract 1.55 µg caffeine/mL extract 82.25 GAE/mL extract	[71]
PS	Total phenolic compounds Caffeine	Ethanol–water: (7:3 *v*/*v*)	50 min 75 °C	Conventional	2.14 g GAE/kg PS 1.34 g caffeine/kg PS	[42]
	Total phenolic compounds	Methanol–HCl (4:1 *v*/*v*)	90 min 100 °C	Conventional	2.3 mg GAE/g PS	[64]
PS	Total phenolic compounds Flavonoids	Water	5 min 5 Hz pulse frequency 6 kV/cm	PEF pretreatment and MAE	321.17 mg GAE/100 g PS 164.328 mg rutin/100 g PS	[63]
	Total phenolic compounds Caffeine Chlorogenic acid Epicatechin	Ethanol	4 h 40 KHz 25 °C	Ultrasonic bath-assisted extraction	2.515 mg GAE/g extract 24.478 mg de caffeine/100 g extract 7.047 mg CGA/100 g extract 1.155 mg de epicatechin/g extract	[38]
	Chlorogenic acid	Ethanol	70 °C 5 min 400 W 2.45 GHz	MAE	7.8 µg CGA/g extract	[72]
SS	Caffeine Chlorogenic acid	NADES (choline chloride–lactic acid–water 1:2:2) Water	30 min 60 °C	Conventional	8.19 mg caffeine/g SS 12.56 mg CGA/g SS 8.04 mg caffeine/mg SS 13.82 mg CGA/g SS	[73]
SS	Total phenolic compounds	Water	30 min 25 °C 38 W/cm^2^	UAE	2.79 mg GAE/g SS	[74]
	Total phenolic compounds Caffeine	Methanol–water (4:1 *v*/*v*)	10 min 60 °C 38 W/cm^2^	UAE	8.94 mg GAE/g SS 4.29 mg caffeine/g SS	[65]
	Lipids Caffeine	Supercritical carbon dioxide (CO_2_)–ethanol (95:5 m/m)	120 min 50 °C 300 Bar	SFE	3.3 g lipids/100 g SS 0.14 g caffeine/g SS	[75]
	Total phenolic compounds Caffeine	Water–ethanol (1:1 *v*/*v*)	60 °C 30 min	Ultrasonic bath-assisted extraction	54.37 µmol GAE/g SS 6.75 mg caffeine/g SS	[76]
	Total phenolic compounds	Water–ethanol (1:1 *v*/*v*) Acetone Ethyl acetate	40 °C 90 min	Conventional	161 mg GAE/L extract 22 mg GAE/L extract 9 mg GAE/L extract	[77]
	Caffeine	Water	20 kHz 600 W 40 °C 10 min	UAE	27.73 mg caffeine/g freeze-dried extract	[78]
SS	Total phenolic compounds	Ethanol–water (63:37 *v*/*v*)	1.37 kV·cm^−1^ 100 A 50 Hz 1000 pulses 25 °C 60 rpm 75 min	PEF pretreatment and conventional	12.12 mg GAE/g SS	[57]
	Total phenolic compounds	Ethanol–water (95:5 m/m) Water Methanol	30 min 60 °C	Conventional	11.2 mg GAE/g extract 9.48 mg GAE/g extract 15.24 mg GAE/g extract	[79]
	Proteins	Sodium hydroxide solution (NaOH) in water 0.6 M	50 °C 200 rpm 10 min 20 kHz 50 °C 10 min 38 W/cm^2^ 434.7 W 10 min	Conventional UAE MAE	6.23% recovered protein 14.04% recovered protein 43.53% recovered protein	[13]
CP	Caffeine	SUPRAS (octanoic acid–ethanol–water)	5 min 25 °C	Conventional	3.6 mg caffeine/g CP	[80]
	Total phenolic compounds	Water	5 min 5 Hz pulse frequency 6 kV/cm	PEF pretreatment and MAE	1433 mg GAE/100 g CP	[63]
	Total phenolic compounds	Ethanol–water (7:3 *v*/*v*)	20 min 40 kHz 25 °C 5 min 70 °C 800 W	Ultrasonic bath-assisted extraction and MAE	328.9 mg GAE/g CP	[45]
	Total phenolic compounds Caffeine Epicatechin	Ethanol	4 h 40 KHz 25 °C	Ultrasonic bath-assisted extraction	12.628 mg GAE/g extract 85.219 mg caffeine/100 g extract 3.458 mg epicatechin/g extract	[38]
	Caffeine	NADES (guanidinium chloride–lactic acid–water 10:17:15) Water	30 min 60 °C	Conventional	8.88 mg caffeine/g CP 8.47 mg caffeine/mg CP	[73]
	Caffeine Chlorogenic acid	NADES (choline chloride–lactic acid–water 1:2:1.5)	60 min 60 °C 200 W	UAE	0.4 g caffeine/100 g CP 0.4 g theobromine/100 g CP	[17]
CP	Total phenolic compounds Flavonoids Chlorogenic acid Caffeine	Water–ethanol (1:1 *v*/*v*)	70 min 700 W 70 °C 35 min 250 W 60 °C	MAE UAE	47 g GAE/g CP 36 g catechin/g CP 8 mg CGA/g CP 6 mg caffeine/g CP 20.86 g GAE/g CP 18.77 g catechin/g CP 2.64 mg CGA/g CP 3.32 mg caffeine/g CP	[81]
CBS	Total phenolic compounds	Water–ethanol (1:1 *v*/*v*)	120 min 25 °C	Conventional	42.97 mg GAE/g CBS	[82]
	Total phenolic compounds	Water	1 min 25 °C	Conventional	8.44 mg GAE/g CBS	[3]
	Total phenolic compounds Flavonoids	Water	15 min 30 bar 150 °C	PLE	37.68 mg GAE/g extract 7.66 g CE/g extract	[83]
	Caffeine Catechin	Water–ethanol (8:2 to 2:8 *v*/*v*) Ethanol	140 °C	PLE	2.5 mg de caffeine/g CBS 5 mg CE/g CBS	[84]
CBS	Total phenolic compounds	Water–ethanol (61:39 *v*/*v*)	1.74 kV·cm^−1^ 100 A 50 Hz 991.28 pulses 25 °C 60 rpm 75 min	PEF pretreatment and conventional	33.05 mg GAE/g CBS	[57]
	Total phenolic compounds	Supercritical carbon dioxide (CO_2_) Ethanol Hexane	2 h 20 MPa 50 °C 11 g/min 20 min 70 °C 10 MPa 6 h	SFE PLE Soxhlet	4.0 mg GAE/g extract 7.2 mg GAE/g extract 5.6 mg GAE/g extract	[85]
	Total phenolic compounds Proteins	Water	90 min 100 °C 5 min 97 °C 500 W 400 rpm 20 °C/min	Conventional MAE	22.1 mg GAE/g CBS 87.0 mg BSA/g CBS 35.9 mg GAE/g CBS 580.0 mg BSA/g CBS	[4]
CBS	Theobromine Catechin	Ethanol	25 min 60, 75 and 90 °C 10.32 MPa	PLE	9.89 mg theobromine /g CBS 3.5 mg CE/g CBS	[86]
	Flavonoids Total phenolic compounds Lipids	Ethanol–water (94:6 m/m)	180 min 90 °C	Conventional	39% total flavanol extraction yield 26.8% total phenolic extraction yield 50% fat extraction yield	[28]
	Total phenolic compounds	NADES (choline chloride–lactic acid 1:2) with 50% of water NADES (choline chloride–glycerol 1:2) with 50% of water NADES (choline chloride–ethylene glycol 1:2) with 50% of water Ethanol–water (7:3 *v*/*v*)	140 min 30 °C	Conventional	14.33 mg GAE/g CBS 11.71 mg GAE/g CBS 11.62 mg GAE/g CBS 9.45 mg GAE/g CBS	[87]
CBS	Total phenolic compounds	Ethanol–water (1:1 *v*/*v*)	2 h 1500 rpm 40 kHz 25 °C	Ultrasonic bath-assisted extraction	21.57 mg GAE/g CBS	[88]
	Total phenolic compounds	Ethanol–water (1:1 *v*/*v*)	15 min 20 kHz 80% amplitude	UAE	263.69 mg GAE/g CBS	[89]
	Proteins Total phenolic compounds	Water	30 min 30 Bar 150 °C	PLE	5.71 g protein/100 g dried extract 37 mg GAE/g extract	[90]
	Total phenolic compounds Flavonoids	Methanol–water–formic acid (70:30:0.1%; *v*/*v*/*v*)	60 min 25 °C	Ultrasonic bath-assisted extraction	42.17 mg GAE/100 mL extract 20.57 mg CE/100 mL extract	[91]
	Caffeine Theobromine	NADES (choline chloride–lactic acid–water 1:2:1.5)	60 min 60 °C 200 W	UAE	0.1 g caffeine/100 g CBS 0.5 g theobromine/100 g CBS	[17]
	Caffeine Theobromine	Water–ethanol (85:15 *v*/*v*)	69 Bar 90 °C	PLE	0.45 mg caffeine/100 g CBS 1.95 mg theobromine/100 g CBS	[92]
CBS	Caffeine Theobromine	NADES (choline chloride–oxalic acid 1:1) with 30% of water	10 min 60 °C 600–800 W	MAE	1.6 mg caffeine/g CBS 5 mg theobromine/g CBS	[93]
	Total phenolic compounds Caffeine	Water	44 min 37 kHz 70 °C 70% ultrasound power	Ultrasonic bath-assisted extraction	118.38 mg GAE/g extract 0.741 mg caffeine/g CBS	[94]
	Total phenolic compounds	Acidified methanol (trifluoroacetic acid 0.3% *v*/*v*)	60 min 38 °C 7 h 60 min 38 °C 20.6 kHz 80 W 30 min 90 °C 21.5 kHz 60 W 60 min 50 or 90 °C 21.5 kHz 60 W	Conventional Soxhlet UAE MAE UAE and MAE at 50 °C UAE and MAE at 90 °C	4.53 mg CE/g CBS 3.54 mg CE/g CBS 15.0 mg CE/g CBS 7.16 mg CE/g CBS 3.53 mg CE/g CBS 4.01 mg CE/g CBS	[95]
CBS	Total phenolic compounds Theobromine	Water	55 min 55 °C 40 kHz 40 min 60 °C 1.5% Viscozyme L	Ultrasonic bath-assisted extraction Enzyme-assisted extraction	3.89 g GAE/100 g CBS 1.03 g theobromine/100 g CBS 3.90 g GAE/100 g CBS 1.29 g theobromine/100 g CBS	[96]

^a^ GAE = gallic acid equivalent; ^b^ CGA = chlorogenic acid equivalent; ^c^ CE = catechin equivalent. CH = cherry husk; CP = coffee pulp; PS = parchment skin; SS = silver skin; CBS = cocoa bean shell. Note: the reader is advised to consult individual references for additional information regarding raw material preparation.

**Table 4:** Main HBDs and HBAs for the formation of NADES used for the extraction of biomolecules.

HBD	HBA	Byproduct	Biomolecule	Reference
Lactic acid Sorbitol	Guanidinium chloride Choline chloride	CP	Caffeine Chlorogenic acid	[73]
Lactic acid	Choline chloride	SS	Caffeine Chlorogenic acid	[73]
Lactic acid	Choline chloride	CBS	Theobromine Caffeine Catechin Epicatechin	[87]
Lactic acid	Choline chloride	CBS CP	Caffeine Theobromine Chlorogenic acid	[17]
Acetamide Butan 1,4-diole Fructose Levulinic acid Malic acid Oxalic acid	Choline chloride	CBS	Galic acid Theobromine Catechin Caffeine Epicatechin	[93]

HBD = hydrogen bond donor; HBA = hydrogen bond acceptor; CH = cherry husk; CP = coffee pulp; PS = parchment skin; SS = silver skin; CBS = cocoa bean shell.

## Data Availability

No new data were created or analyzed in this study. Data sharing is not applicable to this article.
